# Impact of Marital Status, Education, and Family Size on Parental Behaviors Toward Early Childhood Caries in Romania

**DOI:** 10.3390/dj13030111

**Published:** 2025-03-03

**Authors:** Abel Emanuel Moca, Ioan Andrei Țig, Jessica Olivia Cherecheș, Rahela Tabita Moca, Raluca Iurcov

**Affiliations:** Department of Dentistry, Faculty of Medicine and Pharmacy, University of Oradea, 410073 Oradea, Romania; abelmoca@yahoo.com (A.E.M.); rahelamoca@gmail.com (R.T.M.); raluirimie@yahoo.com (R.I.)

**Keywords:** socio-demographic influence, parental KAP, ECC prevention

## Abstract

**Background and Objectives**: This study aimed to examine the influence of socio-demographic factors (marital status, number of children, and education level) on the knowledge, attitudes, and practices (KAP) concerning Early Childhood Caries (ECC) prevention among parents in Bihor, Romania. This research seeks to address the lack of regional data on the influence of socio-demographic factors, such as marital status, number of children, and education level, on parental knowledge, attitudes, and practices regarding ECC prevention. These insights are essential for developing targeted public health interventions in Romania. **Materials and Methods**: Conducted from March to September 2024, this cross-sectional study utilized a KAP questionnaire distributed online to parents of children under six. The survey was adapted to local contexts and included sections on demographic data and ECC-specific knowledge, attitudes, and practices. Statistical analyses, including Chi-square tests, were performed to evaluate the relationship between socio-demographic factors and KAP outcomes, ensuring robust data interpretation under ethical standards set by the Declaration of Helsinki. **Results**: Of the 798 respondents who accessed the questionnaire, 419 completed it, resulting in a completion rate of 52.5%. The participants had a mean age of 33.8 years. In terms of gender distribution, 348 (83.1%) were female and 71 (16.9%) were male. Higher educational levels were strongly correlated with better ECC knowledge and preventive practices; parents with university degrees demonstrated significantly better understanding and engagement in ECC prevention (*p* < 0.05). Married parents participated more actively in ECC prevention than unmarried ones, with 61.1% adhering to recommended practices compared to significantly lower rates among unmarried parents (*p* = 0.020). While this difference was statistically significant, the lower representation of unmarried parents in the sample should be considered when interpreting this finding. Families with fewer children showed more effective ECC preventive practices (*p* = 0.001). **Conclusions**: The study suggests that higher education and smaller family sizes are associated with better ECC prevention behaviors, emphasizing the need for targeted public health interventions. These could include parental education campaigns on ECC prevention, community-based oral health workshops, subsidized fluoride programs, and increased accessibility to pediatric dental services for underprivileged families.

## 1. Introduction

Early Childhood Caries (ECCs) are characterized by the presence of one or more decayed, missing, or filled surfaces in any deciduous tooth of a child under six years of age [1]. This condition exhibits a high global prevalence, affecting approximately 1.76 billion children with primary teeth [2]. ECC is widely recognized as a multifactorial disease influenced by a complex interplay of various factors [2]. Key microbial risk factors in ECC include *Streptococcus mutans* and *Lactobacillus* species, which significantly contribute to early colonization and the progression of dental caries. Recent studies have further explored their interaction within the oral microbiome and their role in cariogenic biofilm formation [3], though emerging pathogens also contribute to its pathogenesis [4] as part of the broader oral microbiome [5].

Diet plays a critical role in the development of ECC, with increased sugar consumption, low intake of fruits and vegetables, and frequent bottle feeding being associated with a higher risk of carious lesions [6]. Additionally, oral hygiene practices among children under six are often inadequate, compounded by challenges faced by parents or caregivers in maintaining proper oral hygiene and a lack of awareness about appropriate measures [7].

If left untreated, ECC can progress rapidly, leading to extensive coronal destruction [8] and adversely affecting the quality of life in children. Symptoms such as dental pain, difficulties with mastication, speech impairments, and social integration issues may arise [9,10]. To mitigate these effects, the International Association of Pediatric Dentistry (IAPD) advocates for dietary restrictions on sugar, regular brushing with fluoride toothpaste (at least 1000 ppm), preventive guidance within the first year of life, and timely dental referrals [1]. Furthermore, the IAPD emphasizes the importance of increasing parental and caregiver awareness of ECC, as misconceptions and inadequate attitudes towards the condition are prevalent [11].

Socio-demographic factors also significantly influence ECC risk. For instance, higher parental education levels are associated with a reduced risk of ECC through better adoption of oro-dental preventive practices [12,13], while larger family sizes and challenging socio-economic conditions are linked to increased risk [14,15]. To assess parental knowledge, attitudes, and practices (KAP) related to ECC, various KAP-type questionnaires have been developed, where K refers to knowledge, A to attitudes or beliefs, and P to practices [16,17].

ECC remains a significant public health concern due to its high prevalence and long-term consequences for children’s oral and overall health [1]. Understanding the socio-demographic determinants of parental knowledge, attitudes, and practices regarding ECC prevention is crucial for designing targeted interventions that can reduce disparities in oral health. By identifying factors such as parental education, marital status, and family size, this study provides valuable insights that can guide health policies and educational programs aimed at improving oral health outcomes in children and, ultimately, the broader population.

In addition, in Romania, there is a notable absence of studies investigating KAP concerning ECC prevention or the impact of socio-demographic variables on ECC prevalence. However, investigating parents’ knowledge, attitudes, and practices toward early childhood caries is vital for effective prevention, targeted interventions, and empowering families to protect children’s oral health.

This study seeks to fill this gap by examining how factors such as parental marital status, level of formal education, and family size affect their KAP regarding ECC in Bihor, Romania. By focusing on prevention strategies, dental visits, treatment of carious lesions in primary teeth, and dietary practices, this study utilized a KAP questionnaire to evaluate the influence of these demographic factors on parents’ understanding and management of ECC. The findings aim to guide targeted public health strategies to reduce ECC incidence and improve oral health outcomes in the region.

## 2. Materials and Methods

### 2.1. Ethical Considerations

This study was conducted in accordance with the ethical guidelines outlined in the 1975 Declaration of Helsinki, with revisions up to the year 2000. Ethical approval was granted by the Research Ethics Committee of the University of Oradea (Approval No. CEFMF/10, issued on 28 February 2024). Participation was entirely voluntary and anonymous, with informed consent implied through the completion of the questionnaire. No financial or material incentives were provided, and respondents were explicitly informed about their right to withdraw at any time.

### 2.2. Participants and Data Collection

This study was designed as a cross-sectional survey conducted over six months, from 1 March 2024 to 1 September 2024. Data collection was performed using an online KAP questionnaire hosted on the digital platform Survio (https://www.survio.com/ro/, last accessed on 3 September 2024), which facilitates web-based survey administration. The questionnaire link was disseminated through targeted social media channels, specifically parent groups on WhatsApp and Facebook (both operated by Meta Platforms, Menlo Park, CA, USA), to maximize participation within the intended demographic.

The KAP questionnaire on ECC was adapted from the framework established by Mani et al. (2012) [16], incorporating modifications tailored to the specific scope of this study. The questionnaire was translated and refined for the Romanian-speaking population to assess parental knowledge, attitudes, and practices related to ECC. It comprised four structured sections:

- Demographic Information: This section gathered socio-demographic details, including gender, age, ethnicity, marital status, residential area, education level, and number of children. The collected data were analyzed to explore potential associations with parental KAP regarding ECC.
- Knowledge Assessment (Items 1–15): Focused on evaluating parents’ awareness of fundamental ECC-related topics, including tooth eruption, fluoride exposure, dietary influences, and recommended dental visits. Responses were categorized as “Yes”, “No”, or “Not sure”.
- Attitude Assessment (Items 16–22): Investigated parental perceptions of ECC prevention, oral hygiene practices, and the importance of routine dental visits. Responses followed a 5-point Likert scale ranging from “Strongly disagree” to “Strongly agree”, with “Not sure” as an alternative option.
- Practice Assessment (Items 23–32): Assessed the frequency of parents’ engagement in preventive oral health behaviors, including toothbrushing routines, dietary management, and pediatric dental visits. Answers were recorded on a 5-point scale (“Never”, “Sometimes”, “Often”, “Always”, and “Not sure”), with higher scores reflecting a stronger commitment to preventive measures.

To ensure participant eligibility, the survey introduction included a screening mechanism requiring respondents to confirm that they were parents or primary caregivers of a child under six years old, residents of Bihor County, and fluent Romanian speakers before proceeding. The system automatically filtered out individuals who did not meet these inclusion criteria. Furthermore, individuals with formal education in dentistry or related fields were informed that they were ineligible to participate, minimizing potential bias.

Exclusion criteria encompassed non-parents or non-primary caregivers of children under six, non-Romanian speakers, non-residents of Bihor County during the study period, incomplete questionnaire submissions, or respondents with professional training in dentistry.

### 2.3. Sample Size Calculation

To determine an adequate sample size for this study, a statistical calculation was performed to ensure a 95% confidence level, corresponding to a Z-score of 1.96, with a 5% margin of error. A 5% margin of error was chosen as it represents a standard threshold in survey-based research, balancing precision and feasibility. The assumed population proportion of 0.5 was selected because it provides the most conservative estimate, ensuring that the sample size is sufficiently large to capture variability within the population, as this value maximizes the required sample size when the true proportion is unknown.

The sample size was computed using the following formula:

$$n=\frac{Z^{2\;}\cdot p\;\cdot (1-p)}{E^{2}}$$

where *Z* represents the critical value for the chosen confidence level, *p* denotes the estimated population proportion, and *E* is the margin of error.

To refine the estimate for a finite population, the sample size was adjusted using the finite population correction formula:

$$n_{adjusted}=\frac{n}{1+\frac{n-1}{N}}$$

where *N* signifies the total population size. Applying this correction, the required minimum sample size for this study was determined to be approximately 377 participants, ensuring statistical representativeness while preserving the desired confidence level and precision of estimates.

### 2.4. Statistical Analysis

Data analysis was conducted using Python (version 3) (Python Software Foundation, Wilmington, DE, USA). Microsoft Excel 2013 (Microsoft Corporation, Redmond, WA, USA) was employed for data management, including organization, sorting, and computation of descriptive statistics such as means and percentages. The final manuscript was drafted and formatted using Microsoft Word 2013.

To examine the relationships between categorical variables, the Chi-square test of independence was utilized. This statistical method was selected for its effectiveness in evaluating associations between categorical data by comparing observed and expected distributions. A *p*-value threshold of 0.05 was applied to determine statistical significance, where values below this cutoff indicated a meaningful association between variables, while non-significant results suggested a lack of strong evidence for dependence.

For instances where the Chi-square test yielded a significant result, post hoc analysis was performed by computing adjusted residuals for each cell within the contingency table. Residual values exceeding ±1.96 were considered statistically relevant, identifying specific category-response patterns that disproportionately influenced the overall Chi-square findings. This approach enabled a more granular interpretation of associations within the dataset.

## 3. Results

### 3.1. Socio-Demographic Characteristics

The online questionnaire received responses from 798 individuals, with 419 completing it, resulting in a completion rate of 52.5%. The mean age of participants was 33.8 years, with 348 (83.1%) females and 71 (16.9%) males. Table 1 presents the distribution of key sociodemographic variables among respondents. Notably, the majority were married (91.9%, *n* = 385), had completed university education (45.9%, *n* = 192), and most reported having one or two children (83.6%, *n* = 350). These findings indicate a demographic profile characterized by a high level of formal education and smaller family sizes.

### 3.2. Marital Status, Formal Education, Number of Children, and Parental Knowledge Regarding ECC

No statistically significant differences were found between marital status and knowledge of Early Childhood Caries (ECCs). However, significant differences were observed based on education for items 2 (*p* = 0.035), 13 (*p* = 0.011), and 14 (*p* = 0.020):

- Item 2: Respondents with a university degree were more likely to agree that “The final permanent tooth typically erupts between ages 11 and 12” (64.6% vs. 50.5% for high school graduates).
- Item 13: A higher percentage of university graduates agreed that “Drinking sweetened beverages from a bottle can cause dental caries” (96.4% vs. 81.0%).
- Item 14: Among respondents with a PhD, 66.8% agreed that “The first visit to the dentist should occur around age 1”, compared to 42.1% of high school graduates.

Significant associations were also found based on the number of children for items 11 (*p* = 0.006) and 14 (*p* = 0.001):

- Item 11: Respondents with four or more children were more likely to disagree that “Breastfeeding can contribute to dental caries” (75.0% vs. 40.0% for those with one child).
- Item 14: Respondents with one child were more likely to agree (65.8%) compared to those with four or more children (25.0%).

Further details on respondents’ characteristics and their responses are presented in Table 2.

### 3.3. Marital Status, Formal Education, Number of Children, and Parental Attitudes Regarding ECC

Table 3 presents parents’ attitudes toward the prevention and treatment of Early Childhood Caries (ECC). Significant differences were noted based on marital status for items 17 (*p* = 0.004), 18 (*p* = 0.020), 20 (*p* < 0.001), 21 (*p* = 0.022), and 22 (*p* < 0.001):

- Item 17: A total of 61.5% of divorced respondents strongly agreed that “sweets can cause cavities”, compared to 48.3% of married.
- Item 18: A total of 76.9% of divorced respondents strongly agreed that brushing is important, more than married (65.7%) and widowed (66.7%) respondents.
- Item 20: Widowed respondents strongly agreed on regular dental visits (66.7%), while divorced respondents showed the lowest strong agreement (23.1%).
- Item 21: A total of 69.2% of divorced respondents strongly agreed that “the dentist’s advice should always be followed”, compared to 66.7% of widowed and 53.0% of married.
- Item 22: Widowed respondents strongly agreed on treating cavities immediately (66.7%), whereas 38.5% of divorced respondents did.

Education also influenced responses for items 16 (*p* = 0.030), 18 (*p* = 0.001), 20 (*p* = 0.020), and 21 (*p* = 0.006):

- Item 16: A total of 41.7% of PhD holders disagreed that “breastfeeding can cause cavities”, compared to 24.2% of high school graduates.
- Item 18: All PhD respondents strongly agreed on the importance of brushing, versus 43.2% of high school graduates.
- Item 20: A total of 50.0% of PhD holders agreed on regular dental visits compared to 45.3% of high school graduates.
- Item 21: Strong agreement for following dentist advice was higher among PhD holders (66.7%) than among high school graduates (37.9%).

Significant associations related to the number of children were found for items 18 (*p* < 0.001), 19 (*p* = 0.005), and 21 (*p* = 0.012):

- Item 18: A total of 70.5% of respondents with one child strongly agreed on brushing importance, compared to 37.5% with four or more children.
- Item 19: Agreement that “fluoride is safe” was higher for those with one child (45.3%) versus those with four or more (16.7%).
- Item 21: Respondents with four or more children showed lower strong agreement on following dentist advice.

Further details on respondents’ characteristics and their responses are provided in Table 3.

### 3.4. Marital Status, Formal Education, Number of Children, and Parental Practices Regarding ECC

Table 4 presents the results for items 23 to 32 on parents’ practices for preventing and treating Early Childhood Caries (ECCs). Significant differences based on marital status were found for item 30, “My child drinks unsweetened milk from a bottle” (*p* = 0.020), with 61.1% of unmarried respondents often giving unsweetened milk, while 66.7% of widowed respondents reported never using it.

For education, item 28—“My child eats sweets only during main meals” (*p* = 0.014)—showed that PhD respondents were more likely to limit sweets to main meals (66.7%) compared to high school graduates (50.5%).

Significant associations based on the number of children were observed for items 27 (*p* = 0.001), 28 (*p* = 0.003), and 30 (*p* = 0.012):

- Item 27: A total of 35.8% of respondents with one child always checked their child’s teeth vs. 12.5% with four or more children.
- Item 28: Those with one child were more likely to always limit sweets (35.8%) compared to those with four or more (12.5%).
- Item 30: A total of 25.8% of one-child families always used unsweetened milk, compared to 8.3% with four or more children.

These findings suggest that marital status and education influence ECC prevention and treatment practices. Detailed results are shown in Table 4.

## 4. Discussion

This study explored the impact of various socio-demographic factors on parental KAP concerning ECC in Bihor, Romania. Our results underscore the significant influence of parental education, marital status, and family size on ECC-related behaviors and beliefs. Given that a child’s hygiene habits are primarily shaped by their parents, who serve as their first role models, these findings highlight the crucial role of caregivers in promoting effective oral hygiene. Children learn the importance and proper technique of tooth brushing by observing and mimicking their caregivers, reinforcing the need for parental education as a key component of ECC prevention programs [19]. The focus of this study was solely on these three demographic characteristics, as aspects related to gender, age, and living environment were previously addressed [18]. Interest in ECC remains high and is increasing due to the alarming prevalence of this pathology globally [20]. Moreover, focusing on the Romanian population is crucial, given the absence of studies that provide a clear overview of both parental KAP regarding ECC and the actual prevalence of this condition in the country.

One of the most significant findings from this study was the impact of marital status on parents’ KAP regarding ECC. Married respondents generally exhibited higher knowledge and more positive attitudes towards ECC prevention compared to their unmarried or divorced counterparts. For instance, married parents were more likely to understand the importance of early dental visits and the role of diet in caries development. In terms of attitudes, married parents more frequently agreed with statements emphasizing the necessity of regular brushing and dental check-ups for their children. This could be attributed to the increased social support and shared responsibility typically associated with marriage, which can facilitate better oral health practices [21,22]. Conversely, single and divorced parents, who often face additional financial and time constraints, showed less consistent ECC-related practices, such as regular dental visits and the use of fluoride toothpaste. Additionally, children of divorced parents have an increased risk of dental caries [23]. The findings of this study regarding the influence of marital status on KAP towards ECC are consistent with other studies in the literature. Al-Jaber et al. (2022) identified that the knowledge of married respondents regarding ECC was higher than that of divorced respondents. However, unlike our study, the percentage of positive attitudes and practices was similar between married and divorced respondents, though these were not statistically significant [17]. These results are also confirmed by the study of Alos-Rullan V. (2019), which found that children from unmarried-parent families were more likely to have fillings due to dental caries and were at a higher risk of developing dental caries [24]. These differences highlight the necessity for tailored interventions that address the unique challenges faced by single-parent households, ensuring that all children, regardless of family structure, have access to optimal oral healthcare.

The study revealed a strong association between parental education levels and their KAP regarding ECC. Respondents with higher educational attainment, such as a university degree or above, demonstrated significantly better knowledge about ECC risk factors, including the impact of sugary foods and the necessity of early dental visits. Specifically, a significant difference was observed for item 14, with PhD holders showing greater awareness of the recommended age for the first dental visit compared to high school graduates. This may be due to increased exposure to the scientific literature and professional healthcare guidelines among highly educated individuals. In contrast, parents with lower education levels may lack access to evidence-based recommendations, leading to delayed dental visits for their children [25]. These parents also held more positive attitudes towards preventive measures and were more consistent in implementing good oral hygiene practices, such as regular brushing with fluoride toothpaste. Conversely, parents with lower educational levels exhibited greater uncertainty and less adherence to recommended ECC prevention strategies. The impact of education on better oral health knowledge, attitudes, and practices has also been identified in other studies. Lower education is a key determinant of poor oral hygiene in children, as it is often associated with limited awareness of preventive measures and reduced access to pediatric dental care. Previous studies have demonstrated that socioeconomically disadvantaged populations, including those with lower education levels, face significant barriers to accessing oral health services [26]. For instance, Al-Jaber et al. (2022) found similar results to the present study, noting that parents with a university degree or higher had a significantly higher percentage of appropriate attitudes toward ECC prevention [17]. Additionally, the study by Mohammed Al-Dahan and Ali Ismael (2023) found that individuals without any formal education had much lower KAP levels compared to those with any level of formal education [27]. These findings are supported by a scoping review from 2024 aimed at mapping the current evidence on the associations between parental education and ECC. The authors of the scoping review highlighted that mothers with lower educational levels introduced sugary drinks and foods early, while those with higher education levels exhibited better preventive behaviors towards their children’s oral health, had children with better oral hygiene indices, less dental plaque, lower levels of *Streptococcus mutans*, and used fluoride toothpaste earlier [12]. In addition, a notable difference was observed in item 16, where 41.7% of PhD holders disagreed with the statement that “breastfeeding can cause cavities”, compared to only 24.2% of high school graduates. This may indicate that individuals with higher education levels rely more on evidence-based guidelines, which suggest that breastfeeding itself is not a direct cause of caries [28]. However, prolonged nighttime breastfeeding, especially without proper oral hygiene, has been associated with an increased risk of ECC in some studies [29]. Given the small number of PhD holders in the sample, this finding should be interpreted with caution. Further research with a larger, more diverse sample is necessary to explore this trend in greater depth. These insights suggest that educational interventions targeting less-educated parents could be crucial for improving oral health outcomes in children.

The study also found that the number of children significantly influences parents’ KAP regarding ECC. Respondents with fewer children generally demonstrated better knowledge and were more likely to engage in preventive practices, such as regular dental check-ups and consistent oral hygiene routines for their children. In contrast, respondents with larger families reported more challenges in maintaining these practices, likely due to increased caregiving demands and resource constraints [30]. One possible explanation for this trend is that parents with only one child may have more time to focus on their child’s oral hygiene routine, allowing for more frequent monitoring. Additionally, first-time parents might be more attentive and cautious about their child’s health, including dental care, whereas parents with multiple children may have to divide their attention among siblings, making it more challenging to consistently check each child’s teeth [31]. These aspects are supported by other authors. For instance, Park et al. (2023) highlighted that larger families are associated with several oral health risk factors and protective practices and that a higher number of children in the family is linked with an increased risk of developing dental caries [14]. A high number of children was also identified as a risk factor for ECC in the systematic review conducted by Lam et al. (2022) [32]. Since larger family sizes are associated with lower adherence to optimal oral hygiene practices, tailored interventions should be considered. Community-based programs that provide parental education on time-efficient oral care routines, group workshops focusing on practical demonstrations, and home-based reinforcement strategies could help improve oral health outcomes in larger families.

The significant association between parental education and ECC-related knowledge and practices underscores the need for targeted educational programs. Health campaigns aimed at increasing awareness about ECC prevention should prioritize parents with lower educational levels and consider the unique challenges faced by single parents and larger families. Dental professionals should adopt a family-centered approach, offering tailored advice and support to families with varying socio-demographic backgrounds.

Furthermore, the findings suggest that incorporating ECC prevention education into prenatal and early childhood health services could be beneficial. Providing parents with the necessary knowledge and resources early on can help establish good oral health habits that persist as children grow. ECC prevention is not only important for the health of primary teeth but also for the health of permanent teeth, which may erupt in an environment dominated by dental caries [33], and for preventing the development of dentomaxillary anomalies [34]. This pathology can have long-term effects that are not only economically demanding, such as orthodontic treatments and the retention of orthodontic treatment outcomes [35,36], but can also negatively impact the quality of life of patients and their families [37].

One of the strengths of this study is its comprehensive assessment of parental KAP regarding ECC using a questionnaire adapted to the Romanian context. The large sample size enhances the generalizability of the findings to the broader population in Bihor County. Several limitations must be acknowledged in this study. A potential limitation of this study is selection bias due to the online distribution of the questionnaire. Participants who responded may have been more health-conscious or more engaged in oral health topics compared to the general population, potentially influencing the reported knowledge, attitudes, and practices. Additionally, the study sample had a disproportionately high percentage of participants with above-average education levels, which may have further contributed to selection bias. More educated individuals are generally more aware of oral health practices, potentially leading to an overestimation of ECC-related knowledge and behaviors. The cross-sectional design precludes causal inferences about the relationships between socio-demographic factors and ECC-related behaviors. Additionally, reliance on self-reported data may introduce bias, as respondents might overestimate their knowledge or adherence to recommended practices. The completion rate of 52.5% indicates that those who responded may not accurately represent the overall population, potentially limiting the generalizability of the findings. However, given the limited data on ECC in Romania, these findings contribute significantly to understanding the issue within this specific context.

Future studies should consider longitudinal designs to explore how changes in socio-demographic factors over time influence ECC-related KAP. Additionally, intervention studies aimed at improving parental knowledge and practices, particularly among lower-educated and larger families, are warranted. Exploring the impact of cultural factors and parental mental health on ECC prevention could also provide valuable insights for developing more effective prevention strategies.

## 5. Conclusions

This study demonstrates that marital status, number of children, and education level significantly influence parental knowledge, attitudes, and practices regarding ECC in Bihor, Romania. Parents with higher education levels exhibit better understanding and more proactive ECC prevention behaviors. Married respondents generally demonstrated a higher engagement in preventive practices compared to their unmarried counterparts and parents with fewer children tend to engage more consistently in preventive oral health practices compared to those with larger families. The findings emphasize the necessity for educational initiatives specifically designed for parents with lower educational backgrounds and larger families to enhance ECC preventive measures. This research suggests that public health strategies should be customized to effectively address these socio-demographic variables, promoting broader community engagement and targeted interventions to reduce ECC prevalence. This study emphasizes the clinical relevance of parental education and socio-demographic factors in shaping children’s oral health. Understanding these influences can guide pediatric dentists in developing targeted preventive strategies to reduce ECC.

## Figures and Tables

**Table 1:** Respondents’ distribution according to investigated variables [18].

Variable	Size	
	No.	Percentage
**Marital Status**		
Not married	18	4.3%
Married	385	91.9%
Divorced	13	3.1%
Widowed	3	0.7%
**Formal Education**		
Middle school	1	0.3%
High school	95	22.4%
Bachelor’s degree	192	45.9%
Master’s degree	119	28.5%
PhD	12	2.9%
**Number of Children**		
1	190	45.4%
2	160	38.2%
3	45	10.7%
4 or more	24	5.7%

**Table 2:** Distribution of respondents by marital status, formal education, and number of children, and their responses to knowledge (K) items.

Answer	Marital Status				Formal Education					Number of Children			
	NM	M	D	W	MS	HS	U	MD	PhD	1	2	3	≥4
**Item 1: Primary teeth typically begin to erupt around the age of 6 months, though they may emerge earlier.**													
Yes	15 (83.3%)	337 (87.5%)	10 (76.9%)	2 (66.7%)	1 (100.0%)	81 (85.2%)	167 (87.0%)	104 (87.4%)	11 (91.7%)	157 (82.6%)	144 (90.0%)	42 (93.3%)	21 (87.5%)
No	1 (5.6%)	28 (7.3%)	3 (23.1%)	1 (33.3%)	0 (0.0%)	7 (7.4%)	16 (8.3%)	10 (8.4%)	0 (0.0%)	23 (12.1%)	7 (4.4%)	2 (4.4%)	1 (4.2%)
Not sure	2 (11.1%)	20 (5.2%)	0 (0.0%)	0 (0.0%)	0 (0.0%)	7 (7.4%)	9 (4.7%)	5 (4.2%)	1 (8.3%)	10 (5.3%)	9 (5.6%)	1 (2.2%)	2 (8.3%)
*p*	0.177				0.953					0.127			
**Item 2: The final permanent tooth typically erupts between the ages of 11 and 12 years.**													
Yes	14 (77.8%)	214 (55.6%)	9 (69.2%)	2 (66.7%)	1 (100.0%)	48 (50.5%)	124 (64.6%)	59 (49.6%)	7 (58.3%)	113 (59.5%)	85 (53.1%)	28 (62.2%)	13 (54.2%)
No	2 (11.1%)	42 (10.9%)	0 (0.0%)	0 (0.0%)	0 (0.0%)	7 (7.4%)	23 (12.0%)	14 (11.8%)	0 (0.0%)	24 (12.6%)	14 (8.8%)	6 (13.3%)	0 (0.0%)
Not sure	2 (11.1%)	129 (33.5%)	4 (30.8%)	1 (33.3%)	0 (0.0%)	40 (42.1%)	45 (23.4%)	46 (38.6%)	5 (41.7%)	53 (27.9%)	61 (38.1%)	11 (24.4%)	11 (45.8%)
*p*	0.390				0.035					0.123			
**Item 3: Dental caries can affect primary teeth immediately after they erupt.**													
Yes	14 (77.8%)	214 (55.6%)	9 (69.2%)	2 (66.7%)	1 (100.0%)	48 (50.5%)	124 (64.6%)	59 (49.6%)	7 (58.3%)	159 (83.7%)	139 (86.9%)	35 (77.8%)	22 (91.7%)
No	1 (5.6%)	21 (5.5%)	0 (0.0%)	0 (0.0%)	0 (0.0%)	7 (7.4%)	6 (3.1%)	8 (6.7%)	1 (8.3%)	13 (6.8%)	3 (1.9%)	6 (13.3%)	0 (0.0%)
Not sure	2 (11.1%)	38 (9.9%)	2 (15.4%)	0 (0.0%)	0 (0.0%)	40 (42.1%)	62 (32.3%)	52 (43.7%)	4 (33.3%)	18 (9.5%)	18 (11.2%)	4 (8.9%)	2 (8.3%)
*p*	0.948				0.393					0.058			
**Item 4: Children may experience pain due to cavities in their primary teeth.**													
Yes	15 (83.3%)	326 (84.7%)	11 (84.6%)	3 (100.0%)	1 (100.0%)	85 (89.5%)	177 (92.2%)	110 (92.4%)	11 (91.7%)	169 (88.9%)	151 (94.4%)	42 (93.3%)	22 (91.7%)
No	0 (0.0%)	5 (1.3%)	0 (0.0%)	0 (0.0%)	0 (0.0%)	2 (2.1%)	3 (1.6%)	0 (0.0%)	0 (0.0%)	3 (1.6%)	2 (1.2%)	0 (0.0%)	0 (0.0%)
Not sure	3 (16.7%)	53 (13.8%)	0 (0.0%)	0 (0.0%)	0 (0.0%)	8 (8.4%)	12 (6.2%)	9 (7.6%)	1 (8.3%)	18 (9.5%)	7 (4.4%)	3 (6.7%)	2 (8.3%)
*p*	0.981				0.929					0.599			
**Item 5: Untreated dental caries can lead to abscesses.**													
Yes	18 (100.0%)	380 (98.7%)	12 (92.3%)	3 (100.0%)	1 (100.0%)	90 (94.7%)	181 (94.3%)	104 (87.4%)	11 (91.7%)	173 (91.1%)	149 (93.1%)	42 (93.3%)	23 (95.8%)
No	0 (0.0%)	10 (2.6%)	0 (0.0%)	0 (0.0%)	0 (0.0%)	0 (0.0%)	1 (0.5%)	0 (0.0%)	0 (0.0%)	1 (0.5%)	0 (0.0%)	0 (0.0%)	0 (0.0%)
Not sure	0 (0.0%)	26 (6.8%)	0 (0.0%)	0 (0.0%)	0 (0.0%)	5 (5.3%)	10 (5.2%)	15 (12.6%)	1 (8.3%)	16 (8.4%)	11 (6.9%)	3 (6.7%)	1 (4.2%)
*p*	0.376				0.441					0.921			
**Item 6: Oral hygiene should be maintained even before the eruption of teeth.**													
Yes	15 (83.3%)	214 (58.2%)	11 (84.6%)	3 (100.0%)	1 (100.0%)	67 (70.5%)	157 (81.8%)	96 (80.7%)	8 (66.6%)	155 (81.6%)	123 (76.9%)	32 (71.1%)	19 (79.2%)
No	1 (5.6%)	117 (30.4%)	2 (15.4%)	0 (0.0%)	0 (0.0%)	10 (10.6%)	15 (7.8%)	9 (7.6%)	2 (16.7%)	15 (7.9%)	18 (11.2%)	3 (6.7%)	0 (0.0%)
Not sure	2 (11.1%)	49 (12.7%)	0 (0.0%)	0 (0.0%)	0 (0.0%)	18 (18.9%)	20 (10.4%)	14 (11.8%)	2 (16.7%)	20 (10.5%)	19 (11.9%)	10 (22.2%)	5 (20.8%)
*p*	0.193				0.530					0.150			
**Item 7: Primary teeth should be brushed from the outset with fluoride toothpaste.**													
Yes	14 (77.8%)	350 (90.6%)	11 (84.6%)	3 (100.0%)	1 (100.0%)	63 (66.4%)	101 (52.6%)	65 (54.6%)	7 (58.3%)	111 (58.4%)	87 (54.4%)	20 (44.4%)	19 (79.2%)
No	0 (0.0%)	20 (5.2%)	0 (0.0%)	0 (0.0%)	0 (0.0%)	18 (18.9%)	69 (35.9%)	42 (35.3%)	3 (25.0%)	55 (28.9%)	54 (33.8%)	21 (46.7%)	2 (8.3%)
Not sure	2 (11.1%)	21 (5.4%)	0 (0.0%)	0 (0.0%)	0 (0.0%)	14 (14.7%)	22 (11.5%)	12 (10.1%)	2 (16.7%)	24 (12.6%)	19 (11.9%)	4 (8.9%)	3 (12.5%)
*p*	0.952				0.209					0.061			
**Item 8: Brushing should be performed twice daily from the beginning.**													
Yes	15 (83.3%)	214 (58.2%)	11 (84.6%)	3 (100.0%)	1 (100.0%)	87 (91.6%)	172 (89.6%)	112 (94.1%)	11 (91.7%)	176 (92.6%)	144 (90.0%)	41 (91.1%)	22 (91.7%)
No	1 (5.6%)	117 (30.4%)	2 (15.4%)	0 (0.0%)	0 (0.0%)	2 (2.1%)	13 (6.8%)	3 (2.5%)	0 (0.0%)	9 (4.7%)	7 (4.4%)	2 (4.4%)	0 (0.0%)
Not sure	2 (11.1%)	49 (12.7%)	0 (0.0%)	0 (0.0%)	0 (0.0%)	6 (6.3%)	7 (3.6%)	4 (3.4%)	1 (8.3%)	5 (2.6%)	9 (5.6%)	2 (4.4%)	2 (8.3%)
*p*	0.150				0.511					0.679			
**Item 9: Parents should brush their child’s teeth, even if the child resists.**													
Yes	14 (77.8%)	350 (90.6%)	11 (84.6%)	3 (100.0%)	1 (100.0%)	87 (91.6%)	177 (92.2%)	109 (91.6%)	11 (91.7%)	173 (91.1%)	148 (92.5%)	41 (91.1%)	23 (95.8%)
No	0 (0.0%)	20 (5.2%)	0 (0.0%)	0 (0.0%)	0 (0.0%)	6 (6.3%)	12 (6.2%)	8 (6.7%)	0 (0.0%)	14 (7.4%)	9 (5.6%)	3 (6.7%)	0 (0.0%)
Not sure	2 (11.1%)	21 (5.4%)	0 (0.0%)	0 (0.0%)	0 (0.0%)	2 (2.1%)	3 (1.6%)	2 (1.7%)	1 (8.3%)	3 (1.6%)	3 (1.9%)	1 (2.2%)	1 (4.2%)
*p*	0.803				0.887					0.828			
**Item 10: The administration of fluoride is an effective method for preventing cavities.**													
Yes	13 (72.2%)	300 (79.2%)	9 (69.2%)	2 (66.7%)	1 (100.0%)	53 (55.8%)	114 (59.4%)	63 (52.9%)	9 (75.0%)	113 (59.5%)	89 (55.6%)	26 (57.8%)	12 (50.0%)
No	1 (5.6%)	35 (9.2%)	2 (15.4%)	0 (0.0%)	0 (0.0%)	8 (8.4%)	22 (11.5%)	15 (12.6%)	1 (8.3%)	20 (10.5%)	17 (10.6%)	8 (17.8%)	1 (4.2%)
Not sure	4 (22.2%)	44 (11.6%)	2 (15.4%)	1 (33.3%)	0 (0.0%)	34 (35.8%)	56 (29.2%)	41 (34.5%)	2 (16.7%)	57 (30.0%)	54 (33.8%)	11 (24.4%)	11 (45.8%)
*p*	0.682				0.760					0.416			
**Item 11: Breastfeeding can contribute to the development of dental caries.**													
Yes	14 (77.8%)	350 (91.1%)	9 (69.2%)	3 (100.0%)	0 (0.0%)	21 (22.1%)	56 (29.2%)	26 (21.8%)	5 (41.7%)	61 (32.1%)	39 (24.4%)	7 (15.6%)	1 (4.2%)
No	1 (5.6%)	18 (4.7%)	3 (23.1%)	0 (0.0%)	1 (100.0%)	48 (50.5%)	85 (44.3%)	64 (53.8%)	6 (50.0%)	76 (40.0%)	85 (53.1%)	25 (55.6%)	18 (75.0%)
Not sure	3 (16.7%)	16 (4.2%)	1 (7.7%)	0 (0.0%)	0 (0.0%)	26 (27.4%)	51 (26.6%)	29 (24.4%)	1 (8.3%)	53 (27.9%)	36 (22.5%)	13 (28.8%)	5 (20.8%)
*p*	0.145				0.491					0.006			
**Item 12: The consumption of sweets can lead to dental caries.**													
Yes	18 (100.0%)	380 (98.7%)	12 (92.3%)	3 (100.0%)	1 (100.0%)	95 (100.0%)	188 (98.0%)	118 (99.2%)	11 (91.7%)	187 (98.4%)	158 (98.8%)	45 (100.0%)	23 (95.8%)
No	0 (0.0%)	10 (2.6%)	0 (0.0%)	0 (0.0%)	0 (0.0%)	0 (0.0%)	2 (1.0%)	1 (0.8%)	0 (0.0%)	1 (0.5%)	2 (1.2%)	0 (0.0%)	0 (0.0%)
Not sure	0 (0.0%)	26 (6.8%)	2 (15.4%)	0 (0.0%)	0 (0.0%)	0 (0.0%)	2 (1.0%)	0 (0.0%)	1 (8.3%)	2 (1.1%)	0 (0.0%)	0 (0.0%)	1 (4.2%)
*p*	0.147				0.122					0.319			
**Item 13: Drinking sweetened beverages from a bottle can cause dental caries.**													
Yes	14 (77.8%)	350 (91.1%)	10 (76.9%)	3 (100.0%)	1 (100%)	77 (81.0%)	185 (96.4%)	105 (88.2%)	10 (83.3%)	170 (89.5%)	145 (90.6%)	41 (91.1%)	22 (91.7%)
No	2 (11.1%)	20 (5.2%)	0 (0.0%)	0 (0.0%)	0 (0.0%)	5 (5.3%)	2 (1.0%)	3 (2.5%)	0 (0.0%)	6 (3.2%)	3 (1.9%)	1 (2.2%)	0 (0.0%)
Not sure	2 (11.1%)	15 (3.9%)	3 (23.1%)	0 (0.0%)	0 (0.0%)	13 (13.7%)	5 (2.6%)	11 (9.3%)	2 (16.7%)	14 (7.4%)	12 (7.5%)	3 (6.7%)	2 (8.3%)
*p*	0.581				0.011					0.971			
**Item 14: The first visit to the dentist should occur around the age of 1 year.**													
Yes	13 (72.2%)	300 (79.2%)	9 (69.2%)	2 (66.7%)	1 (100.0%)	40 (42.1%)	116 (60.4%)	77 (64.7%)	8 (66.8%)	125 (65.8%)	86 (53.8%)	25 (55.6%)	6 (25.0%)
No	3 (16.7%)	36 (9.5%)	1 (7.7%)	0 (0.0%)	0 (0.0%)	11 (11.6%)	26 (13.5%)	12 (10.1%)	2 (16.6%)	21 (11.1%)	22 (13.8%)	7 (15.6%)	1 (4.2%)
Not sure	2 (11.1%)	42 (11.1%)	3 (23.1%)	1 (33.3%)	0 (0.0%)	44 (46.7%)	50 (26.1%)	30 (25.2%)	2 (16.6%)	44 (23.2%)	52 (32.5%)	13 (28.8%)	17 (70.8%)
*p*	0.420				0.020					0.000			
**Item 15: Cavities in primary teeth require immediate treatment upon detection.**													
Yes	15 (83.3%)	320 (84.4%)	10 (76.9%)	3 (100.0%)	1 (100.0%)	77 (81.1%)	152 (79.2%)	86 (72.3%)	10 (83.4%)	137 (72.1%)	129 (80.6%)	39 (86.7%)	21 (87.5%)
No	0 (0.0%)	21 (5.4%)	1 (7.7%)	0 (0.0%)	0 (0.0%)	12 (12.6%)	18 (9.4%)	11 (9.2%)	1 (8.3%)	21 (11.1%)	17 (10.6%)	3 (6.7%)	1 (4.2%)
Not sure	3 (16.7%)	39 (10.2%)	2 (15.4%)	0 (0.0%)	0 (0.0%)	6 (6.3%)	22 (11.5%)	22 (18.5%)	1 (8.3%)	32 (16.8%)	14 (8.8%)	3 (6.7%)	2 (8.3%)
*p*	0.161				0.376					0.141			

NM—not married, M—married, D—divorced, W—widowed, MS—middle school, HS—high school, U—university, MD—master’s degree.

**Table 3:** Distribution of respondents by marital status, formal education, and number of children, and their responses to attitude (A) items.

Answer	Marital Status				Formal Education					Number of Children			
	NM	M	D	W	MS	HS	U	MD	PhD	1	2	3	≥4
**Item 16: I believe that breastfeeding can cause cavities in my child.**													
S. Disagree	4 (22.2%)	91 (23.6%)	2 (15.4%)	0 (0.0%)	0 (0.0%)	27 (28.4%)	36 (18.8%)	32 (26.9%)	2 (16.7%)	48 (25.3%)	39 (24.4%)	6 (13.3%)	4 (16.7%)
Disagree	8 (44.4%)	122 (31.7%)	4 (30.8%)	1 (33.3%)	0 (0.0%)	23 (24.2%)	68 (35.4%)	39 (32.8%)	5 (41.7%)	52 (27.4%)	52 (32.5%)	23 (51.1%)	8 (33.3%)
Agree	3 (16.7%)	60 (15.6%)	2 (15.4%)	0 (0.0%)	0 (0.0%)	8 (8.4%)	38 (19.8%)	16 (13.4%)	3 (25.0%)	34 (17.9%)	26 (16.2%)	4 (8.9%)	1 (4.2%)
S. Agree	0 (0.0%)	3 (0.8%)	1 (7.7%)	0 (0.0%)	0 (0.0%)	1 (1.1%)	1 (0.5%)	1 (0.8%)	1 (8.3%)	2 (1.1%)	2 (1.2%)	0 (0.0%)	0 (0.0%)
Not sure	3 (16.7%)	109 (28.3%)	4 (30.8%)	2 (66.7%)	1 (100.0%)	36 (37.9%)	49 (25.5%)	31 (26.1%)	1 (8.3%)	54 (28.4%)	41 (25.6%)	12 (26.7%)	11 (45.8%)
*p*	0.495				0.030					0.147			
**Item 17: I believe that sweets can cause cavities in my child.**													
S. Disagree	1 (5.6%)	0 (0.0%)	0 (0.0%)	0 (0.0%)	0 (0.0%)	1 (1.1%)	0 (0.0%)	0 (0.0%)	0 (0.0%)	1 (0.5%)	0 (0.0%)	0 (0.0%)	0 (0.0%)
Disagree	1 (5.6%)	6 (1.6%)	0 (0.0%)	0 (0.0%)	0 (0.0%)	1 (1.1%)	4 (2.1%)	2 (1.7%)	0 (0.0%)	5 (2.6%)	2 (1.2%)	0 (0.0%)	0 (0.0%)
Agree	7 (38.9%)	189 (49.1%)	5 (38.5%)	2 (66.7%)	0 (0.0%)	63 (66.3%)	86 (44.8%)	48 (40.3%)	6 (50.0%)	86 (45.3%)	78 (48.8%)	25 (55.6%)	14 (58.3%)
S. Agree	8 (44.4%)	186 (48.3%)	8 (61.5%)	1 (33.3%)	1 (100.0%)	28 (29.5%)	101 (52.6%)	67 (56.3%)	6 (50.0%)	95 (50.0%)	79 (49.4%)	20 (44.4%)	9 (37.5%)
Not sure	1 (5.6%)	4 (1.0%)	0 (0.0%)	0 (0.0%)	0 (0.0%)	2 (2.1%)	1 (0.5%)	2 (1.7%)	0 (0.0%)	3 (1.6%)	1 (0.6%)	0 (0.0%)	1 (4.2%)
*p*	0.004				0.078					0.721			
**Item 18: I believe that brushing is very important for my child.**													
S. Disagree	1 (5.6%)	0 (0.0%)	0 (0.0%)	0 (0.0%)	0 (0.0%)	1 (1.1%)	0 (0.0%)	0 (0.0%)	0 (0.0%)	1 (0.5%)	0 (0.0%)	0 (0.0%)	0 (0.0%)
Disagree	0 (0.0%)	2 (0.5%)	0 (0.0%)	0 (0.0%)	0 (0.0%)	1 (1.1%)	1 (0.5%)	0 (0.0%)	0 (0.0%)	2 (1.1%)	0 (0.0%)	0 (0.0%)	0 (0.0%)
Agree	7 (38.9%)	125 (32.5%)	3 (23.1%)	1 (33.3%)	0 (0.0%)	48 (50.5%)	59 (30.7%)	25 (21.0%)	4 (33.3%)	53 (27.9%)	55 (34.4%)	17 (37.8%)	11 (45.8%)
S. Agree	10 (55.6%)	253 (65.7%)	10 (76.9%)	2 (66.7%)	1 (100.0%)	41 (43.2%)	132 (68.8%)	93 (78.2%)	8 (66.7%)	134 (70.5%)	104 (65.0%)	28 (62.2%)	9 (37.5%)
Not sure	0 (0.0%)	5 (1.3%)	0 (0.0%)	0 (0.0%)	0 (0.0%)	4 (4.2%)	0 (0.0%)	1 (0.8%)	0 (0.0%)	0 (0.0%)	1 (0.6%)	0 (0.0%)	4 (16.7%)
*p*	0.020				0.001					0.000			
**Item 19: I believe that fluoride is a safe method for preventing dental cavities.**													
S. Disagree	1 (5.6%)	6 (1.6%)	0 (0.0%)	0 (0.0%)	0 (0.0%)	2 (2.1%)	2 (1.0%)	3 (2.5%)	0 (0.0%)	4 (2.1%)	2 (1.2%)	0 (0.0%)	1 (4.2%)
Disagree	1 (5.6%)	34 (8.8%)	1 (7.7%)	0 (0.0%)	0 (0.0%)	10 (10.5%)	14 (7.3%)	12 (10.1%)	0 (0.0%)	16 (8.4%)	11 (6.9%)	8 (17.8%)	1 (4.2%)
Agree	7 (38.9%)	158 (41.0%)	6 (46.2%)	1 (33.3%)	1 (100.0%)	37 (38.9%)	79 (41.1%)	52 (43.7%)	3 (25.0%)	86 (45.3%)	65 (40.6%)	17 (37.8%)	4 (16.7%)
S. Agree	3 (16.7%)	45 (11.7%)	2 (15.4%)	0 (0.0%)	0 (0.0%)	8 (8.4%)	29 (15.1%)	8 (6.7%)	5 (41.7%)	27 (14.2%)	19 (11.9%)	4 (8.9%)	0 (0.0%)
Not sure	6 (33.3%)	142 (36.9%)	4 (30.8%)	2 (66.7%)	0 (0.0%)	38 (40.0%)	68 (35.4%)	44 (37.0%)	4 (33.3%)	57 (30.0%)	63 (39.4%)	16 (35.6%)	18 (75.0%)
*p*	0.977				0.198					0.005			
**Item 20: I believe that regular dental visits for my child should be adhered to.**													
S. Disagree	1 (5.6%)	0 (0.0%)	0 (0.0%)	0 (0.0%)	0 (0.0%)	3 (3.2%)	5 (2.6%)	3 (2.6%)	0 (0.0%)	1 (0.5%)	0 (0.0%)	0 (0.0%)	0 (0.0%)
Disagree	0 (0.0%)	3 (0.8%)	0 (0.0%)	0 (0.0%)	0 (0.0%)	5 (5.3%)	13 (6.7%)	6 (5.0%)	0 (0.0%)	2 (1.1%)	1 (0.6%)	0 (0.0%)	0 (0.0%)
Agree	7 (38.9%)	187 (48.6%)	7 (53.8%)	1 (33.3%)	1 (100%)	43 (45.3%)	94 (48.9%)	63 (53.0%)	6 (50.0%)	81 (42.6%)	85 (53.1%)	20 (44.4%)	16 (66.7%)
S. Agree	8 (44.4%)	189 (49.1%)	3 (23.1%)	2 (66.7%)	0 (0.0%)	18 (18.8%)	58 (30.2%)	31 (26.2%)	5 (41.7%)	103 (54.2%)	69 (43.1%)	24 (53.3%)	6 (25.0%)
Not sure	2 (11.1%)	6 (1.6%)	3 (23.1%)	0 (0.0%)	0 (0.0%)	26 (27.4%)	22 (11.6%)	31 (26.2%)	1 (8.3%)	3 (1.6%)	5 (3.1%)	1 (2.2%)	2 (8.3%)
*p*	0.000				0.020					0.240			
**Item 21: I believe that the dentist’s advice regarding my child’s oral health should always be followed.**													
S. Disagree	1 (5.6%)	1 (0.3%)	0 (0.0%)	0 (0.0%)	0 (0.0%)	2 (2.1%)	0 (0.0%)	0 (0.0%)	0 (0.0%)	2 (1.1%)	0 (0.0%)	0 (0.0%)	0 (0.0%)
Disagree	0 (0.0%)	2 (0.5%)	0 (0.0%)	0 (0.0%)	0 (0.0%)	0 (0.0%)	1 (0.5%)	1 (0.8%)	0 (0.0%)	1 (0.5%)	1 (0.6%)	0 (0.0%)	0 (0.0%)
Agree	7 (38.9%)	176 (45.7%)	3 (23.1%)	1 (33.3%)	0 (0.0%)	57 (60.0%)	71 (37.0%)	56 (47.1%)	3 (25.0%)	64 (33.7%)	80 (50.0%)	27 (60.0%)	16 (66.7%)
S. Agree	9 (50.0%)	204 (53.0%)	9 (69.2%)	2 (66.7%)	1 (100.0%)	36 (37.9%)	119 (62.0%)	60 (50.4%)	8 (66.7%)	121 (63.7%)	78 (48.8%)	18 (40.0%)	7 (29.2%)
Not sure	1 (5.6%)	2 (0.5%)	1 (7.7%)	0 (0.0%)	0 (0.0%)	0 (0.0%)	1 (0.5%)	2 (1.7%)	1 (8.3%)	2 (1.1%)	1 (0.6%)	0 (0.0%)	1 (4.2%)
*p*	0.022				0.006					0.012			
**Item 22: I believe that when cavities appear, they should be treated immediately, even in primary teeth.**													
S. Disagree	1 (5.6%)	0 (0.0%)	0 (0.0%)	0 (0.0%)	0 (0.0%)	1 (1.1%)	0 (0.0%)	0 (0.0%)	0 (0.0%)	1 (0.5%)	0 (0.0%)	0 (0.0%)	0 (0.0%)
Disagree	3 (16.7%)	4 (1.0%)	1 (7.7%)	0 (0.0%)	0 (0.0%)	6 (6.3%)	2 (1.0%)	0 (0.0%)	0 (0.0%)	1 (0.5%)	6 (3.8%)	1 (2.2%)	0 (0.0%)
Agree	4 (22.2%)	171 (44.4%)	6 (46.2%)	1 (33.3%)	0 (0.0%)	45 (47.4%)	79 (41.1%)	53 (44.5%)	5 (41.7%)	76 (40.0%)	70 (43.8%)	25 (55.6%)	11 (45.8%)
S. Agree	10 (55.6%)	188 (48.8%)	5 (38.5%)	2 (66.7%)	1 (100.0%)	35 (36.8%)	103 (53.6%)	60 (50.4%)	6 (50.0%)	98 (51.6%)	77 (48.1%)	18 (40.0%)	12 (50.0%)
Not sure	0 (0.0%)	22 (5.7%)	1 (7.7%)	0 (0.0%)	0 (0.0%)	8 (8.4%)	8 (4.2%)	6 (5.0%)	1 (8.3%)	14 (7.4%)	7 (4.4%)	1 (2.2%)	1 (4.2%)
*p*	0.000				0.088					0.440			

NM—not married, M—married, D—divorced, W—widowed, MS—middle school, HS—high school, U—university, MD—master’s degree.

**Table 4:** Distribution of respondents by marital status, formal education, and number of children, and their responses to practice (P) items.

Answer	Marital Status				Formal Education					Number of Children			
	NM	M	D	W	MS	HS	U	MD	PhD	1	2	3	≥4
**Item 23: I always manage to brush my child’s teeth twice a day.**													
Never	0 (0.0%)	7 (1.8%)	1 (7.7%)	0 (0.0%)	0 (0.0%)	3 (3.2%)	5 (2.6%)	0 (0.0%)	0 (0.0%)	3 (1.6%)	3 (1.9%)	1 (2.2%)	1 (4.2%)
Sometimes	1 (5.6%)	90 (23.4%)	2 (15.4%)	0 (0.0%)	0 (0.0%)	23 (24.2%)	38 (19.8%)	31 (26.1%)	1 (8.3%)	41 (21.6%)	37 (23.1%)	10 (22.2%)	5 (20.8%)
Often	14 (77.8%)	207 (53.8%)	7 (53.8%)	2 (66.7%)	0 (0.0%)	56 (58.9%)	106 (55.2%)	63 (52.9%)	5 (41.7%)	94 (49.5%)	94 (58.8%)	27 (60.0%)	15 (62.5%)
Always	2 (11.1%)	58 (15.1%)	2 (15.4%)	1 (33.3%)	1 (100.0%)	7 (7.4%)	30 (15.6%)	20 (16.8%)	5 (41.7%)	40 (21.1%)	18 (11.2%)	3 (6.7%)	2 (8.3%)
Not sure	1 (5.6%)	23 (6.0%)	1 (7.7%)	0 (0.0%)	0 (0.0%)	6 (6.3%)	13 (6.8%)	5 (4.2%)	1 (8.3%)	12 (6.3%)	8 (5.0%)	4 (8.9%)	1 (4.2%)
*p*	0.694				0.106					0.385			
**Item 24: Brushing is done easily, without any protests from my child.**													
Never	2 (11.1%)	9 (2.3%)	0 (0.0%)	0 (0.0%)	0 (0.0%)	3 (3.2%)	3 (1.6%)	5 (4.2%)	0 (0.0%)	8 (4.2%)	1 (0.6%)	2 (4.4%)	0 (0.0%)
Sometimes	3 (16.7%)	105 (27.3%)	2 (15.4%)	0 (0.0%)	0 (0.0%)	19 (20.0%)	52 (27.1%)	36 (30.3%)	3 (25.0%)	57 (30.0%)	36 (22.5%)	11 (24.4%)	6 (25.0%)
Often	10 (55.6%)	168 (43.6%)	8 (61.5%)	1 (33.3%)	0 (0.0%)	46 (48.4%)	89 (46.4%)	47 (39.5%)	5 (41.7%)	78 (41.1%)	79 (49.4%)	21 (46.7%)	9 (37.5%)
Always	2 (11.1%)	85 (22.1%)	3 (23.1%)	2 (66.7%)	1 (100.0%)	21 (22.1%)	39 (20.3%)	27 (22.7%)	4 (33.3%)	36 (18.9%)	38 (23.8%)	10 (22.2%)	8 (33.3%)
Not sure	1 (5.6%)	18 (4.7%)	0 (0.0%)	0 (0.0%)	0 (0.0%)	6 (6.3%)	9 (4.7%)	4 (3.4%)	0 (0.0%)	11 (5.8%)	6 (3.8%)	1 (2.2%)	1 (4.2%)
*p*	0.326				0.755					0.375			
**Item 25: I use fluoride toothpaste for my child.**													
Never	0 (0.0%)	32 (8.3%)	0 (0.0%)	0 (0.0%)	0 (0.0%)	7 (7.4%)	13 (6.8%)	12 (10.1%)	0 (0.0%)	20 (10.5%)	6 (3.8%)	6 (13.3%)	0 (0.0%)
Sometimes	1 (5.6%)	56 (14.5%)	1 (7.7%)	1 (33.3%)	0 (0.0%)	11 (11.6%)	22 (11.5%)	22 (18.5%)	4 (33.3%)	27 (14.2%)	21 (13.1%)	7 (15.6%)	4 (16.7%)
Often	13 (72.2%)	161 (41.8%)	7 (53.8%)	0 (0.0%)	0 (0.0%)	43 (45.3%)	91 (47.4%)	44 (37.0%)	3 (25.0%)	75 (39.5%)	71 (44.4%)	18 (40.0%)	17 (70.8%)
Always	3 (16.7%)	87 (22.6%)	3 (23.1%)	1 (33.3%)	1 (100.0%)	21 (22.1%)	43 (22.4%)	24 (20.2%)	5 (41.7%)	44 (23.2%)	39 (24.4%)	10 (22.2%)	1 (4.2%)
Not sure	1 (5.6%)	49 (12.7%)	2 (15.4%)	1 (33.3%)	0 (0.0%)	13 (13.7%)	23 (12.0%)	17 (14.3%)	0 (0.0%)	24 (12.6%)	23 (14.4%)	4 (8.9%)	2 (8.3%)
*p*	0.403				0.284					0.079			
**Item 26: I take my child to the dentist for regular check-ups.**													
Never	0 (0.0%)	7 (1.8%)	0 (0.0%)	0 (0.0%)	0 (0.0%)	5 (5.3%)	1 (0.5%)	1 (0.8%)	0 (0.0%)	4 (2.1%)	3 (1.9%)	0 (0.0%)	0 (0.0%)
Sometimes	0 (0.0%)	46 (11.9%)	4 (30.8%)	1 (33.3%)	0 (0.0%)	13 (13.7%)	22 (11.5%)	15 (12.6%)	1 (8.3%)	18 (9.5%)	22 (13.8%)	8 (17.8%)	3 (12.5%)
Often	11 (61.1%)	189 (49.1%)	7 (53.8%)	0 (0.0%)	0 (0.0%)	52 (54.7%)	100 (52.1%)	51 (42.9%)	4 (33.3%)	85 (44.7%)	82 (51.2%)	23 (51.1%)	17 (70.8%)
Always	7 (38.9%)	130 (33.8%)	2 (15.4%)	2 (66.7%)	1 (100.0%)	23 (24.2%)	62 (32.3%)	48 (40.3%)	7 (58.3%)	76 (40.0%)	49 (30.6%)	12 (26.7%)	4 (16.7%)
Not sure	0 (0.0%)	13 (3.4%)	0 (0.0%)	0 (0.0%)	0 (0.0%)	2 (2.1%)	7 (3.6%)	4 (3.4%)	0 (0.0%)	7 (3.7%)	4 (2.5%)	2 (4.4%)	0 (0.0%)
*p*	0.353				0.154					0.297			
**Item 27: I regularly check my child’s teeth.**													
Never	0 (0.0%)	4 (1.0%)	0 (0.0%)	0 (0.0%)	0 (0.0%)	2 (2.1%)	0 (0.0%)	2 (1.7%)	0 (0.0%)	2 (1.1%)	1 (0.6%)	1 (2.2%)	0 (0.0%)
Sometimes	0 (0.0%)	60 (15.6%)	1 (7.7%)	0 (0.0%)	0 (0.0%)	14 (14.7%)	29 (15.1%)	18 (15.1%)	0 (0.0%)	18 (9.5%)	27 (16.9%)	12 (26.7%)	4 (16.7%)
Often	15 (83.3%)	204 (53.0%)	9 (69.2%)	2 (66.7%)	0 (0.0%)	58 (61.1%)	103 (53.6%)	62 (52.1%)	7 (58.3%)	99 (52.1%)	94 (58.8%)	21 (46.7%)	16 (66.7%)
Always	3 (16.7%)	99 (25.7%)	3 (23.1%)	1 (33.3%)	1 (100.0%)	18 (18.9%)	46 (24.0%)	36 (30.3%)	5 (41.7%)	68 (35.8%)	27 (16.9%)	8 (17.8%)	3 (12.5%)
Not sure	0 (0.0%)	18 (4.7%)	0 (0.0%)	0 (0.0%)	0 (0.0%)	3 (3.2%)	14 (7.3%)	1 (0.8%)	0 (0.0%)	3 (1.6%)	11 (6.9%)	3 (6.7%)	1 (4.2%)
*p*	0.631				0.150					0.001			
**Item 28: My child eats sweets only during main meals.**													
Never	1 (5.6%)	56 (14.5%)	4 (30.8%)	1 (33.3%)	0 (0.0%)	18 (18.9%)	27 (14.1%)	16 (13.4%)	1 (8.3%)	38 (20.0%)	19 (11.9%)	1 (2.2%)	4 (16.7%)
Sometimes	8 (44.4%)	188 (48.8%)	5 (38.5%)	2 (66.7%)	0 (0.0%)	48 (50.5%)	83 (43.2%)	64 (53.8%)	8 (66.7%)	73 (38.4%)	94 (58.8%)	23 (51.1%)	13 (54.2%)
Often	4 (22.2%)	88 (22.9%)	2 (15.4%)	0 (0.0%)	0 (0.0%)	14 (14.7%)	52 (27.1%)	28 (23.5%)	0 (0.0%)	50 (26.3%)	28 (17.5%)	14 (31.1%)	2 (8.3%)
Always	1 (5.6%)	17 (4.4%)	1 (7.7%)	0 (0.0%)	1 (100.0%)	4 (4.2%)	8 (4.2%)	4 (3.4%)	2 (16.7%)	12 (6.3%)	5 (3.1%)	2 (4.4%)	0 (0.0%)
Not sure	4 (22.2%)	36 (9.4%)	1 (7.7%)	0 (0.0%)	0 (0.0%)	11 (11.6%)	22 (11.5%)	7 (5.9%)	1 (8.3%)	17 (8.9%)	14 (8.8%)	5 (11.1%)	5 (20.8%)
*p*	0.679				0.001					0.003			
**Item 29: My child is still breastfed.**													
Never	2 (11.1%)	159 (41.3%)	7 (53.8%)	2 (66.7%)	0 (0.0%)	48 (50.5%)	67 (34.9%)	50 (42.0%)	5 (41.7%)	71 (37.4%)	69 (43.1%)	17 (37.8%)	13 (54.2%)
Sometimes	2 (11.1%)	69 (17.9%)	1 (7.7%)	0 (0.0%)	1 (100.0%)	12 (12.6%)	37 (19.3%)	18 (15.1%)	4 (33.3%)	39 (20.5%)	27 (16.9%)	5 (11.1%)	1 (4.2%)
Often	10 (55.6%)	86 (22.3%)	4 (30.8%)	0 (0.0%)	0 (0.0%)	22 (23.2%)	49 (25.5%)	26 (21.8%)	3 (25.0%)	42 (22.1%)	40 (25.0%)	13 (28.9%)	5 (20.8%)
Always	4 (22.2%)	61 (15.8%)	1 (7.7%)	1 (33.3%)	0 (0.0%)	9 (9.5%)	35 (18.2%)	23 (19.3%)	0 (0.0%)	33 (17.4%)	19 (11.9%)	10 (22.2%)	5 (20.8%)
Not sure	0 (0.0%)	10 (2.6%)	0 (0.0%)	0 (0.0%)	0 (0.0%)	4 (4.2%)	4 (2.1%)	2 (1.7%)	0 (0.0%)	5 (2.6%)	5 (3.1%)	0 (0.0%)	0 (0.0%)
*p*	0.114				0.189					0.382			
**Item 30: My child drinks unsweetened milk from a bottle.**													
Never	0 (0.0%)	157 (40.8%)	5 (38.5%)	2 (66.7%)	0 (0.0%)	44 (46.3%)	63 (32.8%)	54 (45.4%)	3 (25.0%)	54 (28.4%)	79 (49.4%)	18 (40.0%)	13 (54.2%)
Sometimes	2 (11.1%)	54 (14.0%)	0 (0.0%)	0 (0.0%)	0 (0.0%)	11 (11.6%)	30 (15.6%)	12 (10.1%)	3 (25.0%)	26 (13.7%)	18 (11.2%)	7 (15.6%)	5 (20.8%)
Often	11 (61.1%)	93 (24.2%)	5 (38.5%)	0 (0.0%)	0 (0.0%)	21 (22.1%)	55 (28.6%)	31 (26.1%)	2 (16.7%)	57 (30.0%)	36 (22.5%)	13 (28.9%)	3 (12.5%)
Always	5 (27.8%)	72 (18.7%)	2 (15.4%)	1 (33.3%)	1 (100.0%)	15 (15.8%)	42 (21.9%)	19 (16.0%)	3 (25.0%)	49 (25.8%)	23 (14.4%)	6 (13.3%)	2 (8.3%)
Not sure	0 (0.0%)	9 (2.3%)	1 (7.7%)	0 (0.0%)	0 (0.0%)	4 (4.2%)	2 (1.0%)	3 (2.5%)	1 (8.3%)	4 (2.1%)	4 (2.5%)	1 (2.2%)	1 (4.2%)
*p*	0.020				0.196					0.012			
**Item 31: My child drinks sweetened beverages from a bottle (tea, milk).**													
Never	7 (38.9%)	251 (65.2%)	9 (69.2%)	3 (100.0%)	1 (100.0%)	55 (57.9%)	121 (63.0%)	85 (71.4%)	8 (66.7%)	120 (63.2%)	107 (66.9%)	27 (60.0%)	16 (66.7%)
Sometimes	7 (38.9%)	91 (23.6%)	2 (15.4%)	0 (0.0%)	0 (0.0%)	23 (24.2%)	51 (26.6%)	23 (19.3%)	3 (25.0%)	48 (25.3%)	34 (21.2%)	13 (28.9%)	5 (20.8%)
Often	3 (16.7%)	26 (6.8%)	0 (0.0%)	0 (0.0%)	0 (0.0%)	10 (10.5%)	10 (5.2%)	9 (7.6%)	0 (0.0%)	12 (6.3%)	12 (7.5%)	4 (8.9%)	1 (4.2%)
Always	1 (5.6%)	9 (2.3%)	0 (0.0%)	0 (0.0%)	0 (0.0%)	5 (5.3%)	4 (2.1%)	1 (0.8%)	0 (0.0%)	6 (3.2%)	4 (2.5%)	0 (0.0%)	0 (0.0%)
Not sure	0 (0.0%)	8 (2.1%)	2 (15.4%)	0 (0.0%)	0 (0.0%)	2 (2.1%)	6 (3.1%)	1 (0.8%)	1 (8.3%)	4 (2.1%)	3 (1.9%)	1 (2.2%)	2 (8.3%)
*p*	0.069				0.484					0.773			
**Item 32: If I notice cavities or other oral issues in my child, I immediately schedule an appointment with the dentist.**													
Never	0 (0.0%)	4 (1.0%)	0 (0.0%)	0 (0.0%)	0 (0.0%)	2 (2.1%)	0 (0.0%)	2 (1.7%)	0 (0.0%)	0 (0.0%)	4 (2.5%)	0 (0.0%)	0 (0.0%)
Sometimes	0 (0.0%)	14 (3.6%)	1 (7.7%)	0 (0.0%)	0 (0.0%)	7 (7.4%)	6 (3.1%)	2 (1.7%)	0 (0.0%)	6 (3.2%)	5 (3.1%)	2 (4.4%)	2 (8.3%)
Often	8 (44.4%)	120 (31.2%)	4 (30.8%)	0 (0.0%)	1 (100.0%)	29 (30.5%)	66 (34.4%)	34 (28.6%)	2 (16.7%)	61 (32.1%)	47 (29.4%)	19 (42.2%)	5 (20.8%)
Always	10 (55.6%)	236 (61.3%)	8 (61.5%)	3 (100.0%)	0 (0.0%)	52 (54.7%)	116 (60.4%)	79 (66.4%)	10 (83.3%)	118 (62.1%)	99 (61.9%)	23 (51.1%)	17 (70.8%)
Not sure	0 (0.0%)	11 (2.9%)	0 (0.0%)	0 (0.0%)	0 (0.0%)	5 (5.3%)	4 (2.1%)	2 (1.7%)	0 (0.0%)	5 (2.6%)	5 (3.1%)	1 (2.2%)	0 (0.0%)
*p*	0.942				0.272					0.370			

NM—not married, M—married, D—divorced, W—widowed, MS—middle school, HS—high school, U—university, MD—master’s degree.

## Data Availability

The data presented in this study are available upon request from the corresponding authors. The data are not publicly available due to privacy reasons.
